# Supplementary material for: Exciton-driven antiferromagnetic metal in a correlated van der Waals insulator
Source: Nat Commun. 2021 Aug 10;12:4837. doi: 10.1038/s41467-021-25164-8 (PMC8355133; doi:10.1038/s41467-021-25164-8)
Supplement: Supplementary file 1 — Supplementary Information [file 41467_2021_25164_MOESM1_ESM.pdf]

Supplementary Information for "Exciton-driven antiferromagnetic metal in a correlated van der Waals insulator"

Carina A. Belvin[1,†], Edoardo Baldini[1,†], Ilkem Ozge Ozel[1], Dan Mao[1], Hoi Chun Po[1], Clifford J. Allington[1], Suhan Son[2,3], Beom Hyun Kim[4], Jonghyeon Kim[5], Inho Hwang[2,3], Jae Hoon Kim[5], Je-Geun Park[2,3,*], T. Senthil[1], and Nuh Gedik[1,*]

[1]*Department of Physics, Massachusetts Institute of Technology, Cambridge, Massachusetts 02139, USA*

[2]*Center for Correlated Electron Systems, Institute for Basic Science, Seoul 08826, Korea*

[3]*Center for Quantum Materials, Department of Physics and Astronomy, Seoul National University, Seoul 08826, Korea*

[4]*Korea Institute for Advanced Study, Seoul 02455, Korea*

[5]*Department of Physics, Yonsei University, Seoul 03722, Korea*

[†]*These authors contributed equally to this work.*

[*]*Email: gedik@mit.edu, jgpark10@snu.ac.kr*

Contents

Supplementary Note 1: $NiPS_3$ single crystal characterization **4**

Magnetic susceptibility and heat capacity to characterize the Néel temperature.

Supplementary Note 2: Data analysis procedure for the ultrafast spectrally-resolved THz transmission measurement **5**

Details of the transfer matrix method used to extract the pump-induced changes in the optical properties.

Supplementary Note 3: Experimental details of the ultrafast measurements **10**

A. Photoexcitation scheme 10

Extended optical absorption spectrum of $NiPS_3$ demonstrates that our pump photon energy lies well below the charge-transfer gap.

B. Rise time of the pump-probe traces 11

Comparison to a high-resistivity silicon test sample shows that the rise time of the $NiPS_3$ pump-probe signal is resolution limited.

Supplementary Note 4: Nature of the Drude response **11**

Determination of quantitative properties of the conducting state, discussion of recombination dynamics of the itinerant carriers, and evidence against the scenario of large polarons.

Supplementary Note 5: Mechanism of the coherent magnon generation **19**

A. Pump polarization dependence 19

Lack of a pump polarization dependence rules out conventional magnon generation mechanisms.

B. Pump fluence dependence of the magnon energy 19

Evidence that the coherent magnon generation is non-thermal.

C. THz transmission and THz emission comparison 21

Verification that the magnon oscillations are identical in the transmission and emission signals.

Supplementary Note 6: Significance of our non-equilibrium results and comparison to band semiconductors **22**

Discussion of how our findings are distinct from those of band semiconductors as well as Mott insulators pumped above the gap.

Supplementary Note 7: Homogeneity of the photoinduced state **24**

Evidence against the scenario of phase separation demonstrates the coexistence of itinerant carriers and long-range antiferromagnetism.

Supplementary Note 8: $MnPS_3$ and $FePS_3$ **28**

Comparison of the THz response in closely related van der Waals antiferromagnets.

Supplementary Note 9: Theoretical calculations **29**

Details of the XXZ Hamiltonian of $NiPS_3$ and the symmetries of the zigzag antiferromagnetic ground state. Determination of the spin precession of the lowest-energy zone-center magnon and calculation of the magnon dispersion. Discussion about the redshift of the magnon energy upon photoexcitation.

A. Model Hamiltonian for $NiPS_3$ 29

B. Classical ground state 30

C. Symmetries of the zigzag order 31

D. Magnon dispersion 32

E. Effect of photoexcitation on the magnon energy 40

Supplementary Note 1: $NiPS_3$ single crystal characterization

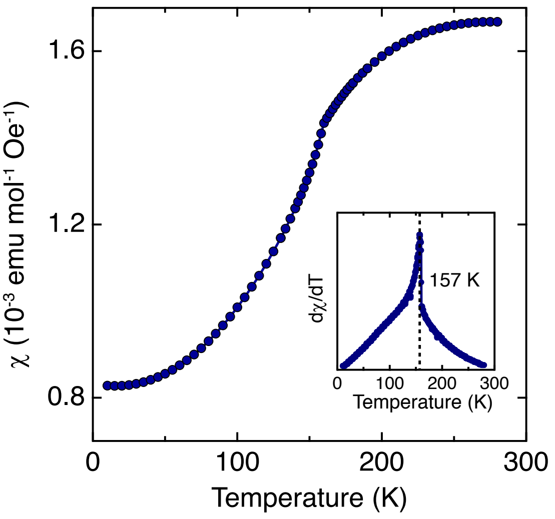

Supplementary Fig. 1 Magnetic susceptibility of our $NiPS_3$ crystal. In-plane magnetic susceptibility χ as a function of temperature. The inset shows its derivative with respect to temperature, with the anomaly indicating that $T_N \sim 157$ K for this crystal.

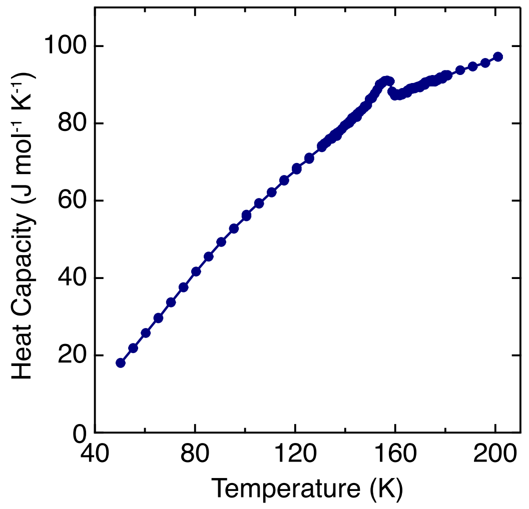

Supplementary Fig. 2 Heat capacity of our $NiPS_3$ crystal. Heat capacity as a function of temperature. The curve shows an anomaly at T_N like the magnetic susceptibility.

Supplementary Note 2: Data analysis procedure for the ultrafast spectrally-resolved THz transmission measurement

In order to fully characterize the low-energy response of $NiPS_3$ to photoexcitation at 1.55 eV, we measure the complete time- and frequency-resolved change in the THz transmission (commonly referred to as a "two-dimensional (2D) scan"). The experimental details of this technique are presented in the Methods section. Here, we describe our data analysis procedure for extracting the pump-induced changes in the optical properties of $NiPS_3$.

The most general approach for obtaining the complex optical parameters from a THz transmission experiment is the transfer matrix method[1,2]. This method calculates the transmission coefficient through a multilayer structure as a product of matrices. The transfer matrix of light at normal incidence passing through the jth layer with complex refractive index $\tilde{n}_j$ and thickness d_j is given by

$$M_j = \begin{pmatrix} \cos\left(\frac{\omega \tilde{n}_j d_j}{c}\right) & -\frac{i}{\tilde{n}_j}\sin\left(\frac{\omega \tilde{n}_j d_j}{c}\right) \\ -i\tilde{n}_j \sin\left(\frac{\omega \tilde{n}_j d_j}{c}\right) & \cos\left(\frac{\omega \tilde{n}_j d_j}{c}\right) \end{pmatrix}, \tag{1}$$

where c is the speed of light. To obtain the total transfer matrix of the system, we take the product of the transfer matrices of each individual layer:

$$M_{\text{tot}} = \prod_{j=1}^{N} M_j = \begin{pmatrix} m_{11} & m_{12} \\ m_{21} & m_{22} \end{pmatrix}, \tag{2}$$

where N is the total number of layers in the structure. We can then derive the transmission coefficient of the system in terms of the matrix elements of M_{tot}:

$$t = \frac{2\tilde{n}_i}{\tilde{n}_i m_{11} + \tilde{n}_i \tilde{n}_f m_{12} + m_{21} + \tilde{n}_f m_{22}}, \tag{3}$$

where the indices i and f refer to the initial and final layers of the stack.

In our ultrafast THz measurement, we detect the THz electric field transmitted through the photoexcited sample ($E_{\text{pump}}(t)$) and compare it to that transmitted through the sample in equilibrium ($E_0(t)$) when the pump pulse is blocked by the optical chopper. By taking the difference $\Delta E(t) = E_{\text{pump}}(t) - E_0(t)$ and performing a Fourier transform, we can relate these measured quantities to the change in the complex transmission coefficient by

$$\frac{\Delta E(\omega)}{E_0(\omega)} = \frac{t_{\text{pump}}(\omega) - t_0(\omega)}{t_0(\omega)}, \tag{4}$$

where $t_{\text{pump}}(\omega)$ and $t_0(\omega)$ are the transmission coefficients through the photoexcited and equilibrium samples, respectively. From this, we obtain the measured change in the transmission coefficient.

The next step is to calculate the transmission through the photoexcited and equilibrium samples using the transfer matrix method as described above. The photoinduced change in the complex refractive index of the material is expected to decay exponentially in the direction of light propagation z as $\tilde{n}(\omega, z) = \tilde{n}_0(\omega) + \Delta\tilde{n}(\omega)e^{-z/d_p}$, where $\tilde{n}_0(\omega)$ is the index of the sample in equilibrium and d_p is the penetration depth of the pump beam. Therefore, the sample can be partitioned into N homogeneous layers of thickness d with refractive index $\tilde{n}(\omega, z)$ determined by the distance z of each layer. Applying the transfer matrix method, one can calculate the change in the transmission coefficient, and by comparing this to the measured value, the change in the complex refractive index $\Delta\tilde{n}(\omega) = \Delta n(\omega) - i\Delta\kappa(\omega)$ can be extracted for each frequency in the THz probe spectrum. Throughout this analysis, we assume that the magnetic contribution to the refractive index is small so that we can determine quantitative parameters of the Drude response. Therefore, from $\Delta\tilde{n}(\omega)$ we can obtain the photoinduced changes

in other relevant optical parameters such as the absorption coefficient $\Delta\alpha(\omega)$ and the complex optical conductivity $\Delta\sigma(\omega) = \Delta\sigma_1(\omega) + i\Delta\sigma_2(\omega)$.

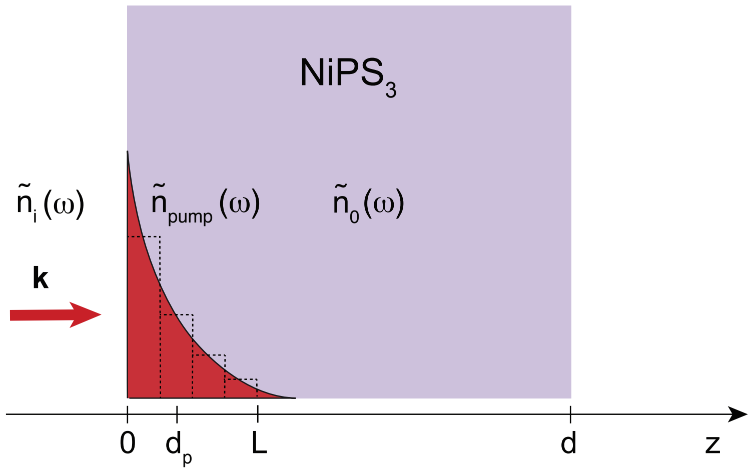

Supplementary Fig. 3 Illustration of the length scales involved in our ultrafast THz transmission experiment. The sample thickness is d = 1.2 mm and the penetration depth of the 1.55 eV pump pulse is d_p = 4.28 μm. We use a total thickness $L = 3d_p$ to represent the photoexcited region, which is partitioned into 4 layers (black dashed rectangles) and has a complex refractive index $\tilde{n}_{\text{pump}}(\omega)$. The complex index of the sample in equilibrium is denoted by $\tilde{n}_0(\omega)$. The light propagates through the multilayer stack in the z direction at normal incidence starting from air ($\tilde{n}_i(\omega) = 1$).

In the case of our ultrafast THz transmission experiment on $NiPS_3$, the penetration depth of the 1.55 eV pump pulse is d_p = 4.28 μm, whereas the sample thickness is d =1.2 mm and the penetration depth of the THz probe beam is $>$ 2 mm for all frequencies in our THz spectrum. A common approximation that is made in ultrafast THz transmission measurements is to treat the photoexcited region as a single, homogeneous layer of thickness d_p[3,4]. However, a recent study that analyzed various approximations used in ultrafast THz measurements found that one should be cautious when applying such approximations as the calculated optical quantities can deviate significantly from

the actual ones[2]. Therefore, we analyzed our data with the full transfer matrix method by partitioning the photoexcited region into multiple layers. We used a total thickness of $L = 3d_p = 12.84$ μm (corresponding to the distance at which the photoinduced change in the index drops to e^{-3}, i.e. 0.05 times its initial value) to represent the photoexcited region rather than the entire sample thickness, as the sample thickness is much larger than d_p and it becomes computationally costly to perform the transfer matrix calculation for a large number of layers. Supplementary Fig. 3 depicts a diagram of our sample dimensions including the photoexcited region (with index $\tilde{n}_{pump}(\omega)$) and the unexcited part of the sample (with index $\tilde{n}_0(\omega)$, which was determined from our equilibrium time-domain THz spectroscopy measurement). The photoinduced change in the index is given by $\Delta\tilde{n}(\omega) = \tilde{n}_{pump}(\omega) - \tilde{n}_0(\omega)$. The transmission coefficient through the sample following photoexcitation is

$$t_{\text{pump}}(\omega) = \frac{2}{m_{11} + \tilde{n}_0 m_{12} + m_{21} + \tilde{n}_0 m_{22}}. \tag{5}$$

The matrix elements are calculated from

$$M_{\text{tot}} = \begin{pmatrix} m_{11} & m_{12} \\ m_{21} & m_{22} \end{pmatrix} = \prod_{j=1}^{N} \begin{pmatrix} \cos\left(\frac{\omega\tilde{n}_j d_j}{c}\right) & -\frac{i}{\tilde{n}_j}\sin\left(\frac{\omega\tilde{n}_j d_j}{c}\right) \\ -i\tilde{n}_j \sin\left(\frac{\omega\tilde{n}_j d_j}{c}\right) & \cos\left(\frac{\omega\tilde{n}_j d_j}{c}\right) \end{pmatrix}, \tag{6}$$

where $\tilde{n}_j = \tilde{n}_0 + \Delta\tilde{n}e^{-(j-0.5)d_j/d_p}$ (we use the z value at the center of each layer) and $d_j = L/N = 3d_p/N$. The transmission coefficient in the absence of the pump pulse is given by

$$t_0(\omega) = \frac{2}{1+\tilde{n}_0} e^{-i\omega\tilde{n}_0 L/c}. \tag{7}$$

To determine the number of layers N needed to obtain a solution with minimal error, we computed $\Delta\sigma_1(\omega)$ at a pump-probe delay of $t = 1.6$ ps (the time delay at which the

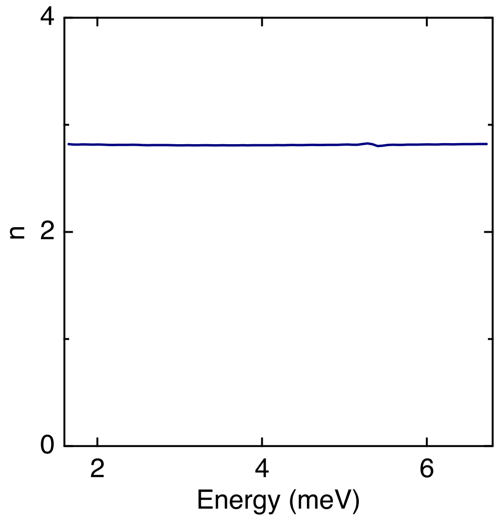

Supplementary Fig. 4 Equilibrium refractive index of NiPS$_3$. Real part of the refractive index in equilibrium as a function of frequency measured by time-domain THz spectroscopy. The index shows almost no frequency dependence throughout the measured spectrum.

Drude response is a maximum - see Fig. 2c in the main text) for several values of N. We found that the extracted optical parameters converge rapidly with increasing values of N. Given this and the fact that it is computationally time consuming to analyze the entire 2D map for large values of N, we have used $N = 4$ to produce the plots in Fig. 2 in the main text. We note that the real part of the refractive index in equilibrium (n_0) is nearly constant across the measured THz spectrum (Supplementary Fig. 4) and it exhibits almost no change upon photoexcitation.

When determining the photoinduced changes in the optical parameters of the system, it is very important to carefully take into account the mismatch between the penetration depths of the pump and probe and their relation to the THz wavelengths over the entire probe spectrum. If we neglect the penetration depth mismatch and instead assume that the pump excitation is uniform across the entire sample thickness, we find

that this analysis underestimates $\Delta\sigma_1$ by two orders of magnitude.

We note that all of the above analysis can only be applied to measure the quasi-steady state response of the system, i.e. pump-induced changes that vary on a timescale that is slow compared to the duration of the THz pulse (~ 1 ps). This is indeed the situation in $NiPS_3$, where the non-equilibrium Drude response and redshifted magnon persist for more than 15 ps (see Fig. 2d in the main text). Moreover, the presence of coherent magnon oscillations has been independently verified in our THz emission measurements (see Supplementary Note 5C). When analyzing the dynamics of a system at early pump probe delays (< 1 ps), one needs to utilize a more involved approach, such as the finite-difference time-domain method[5], which takes into account the response function of the experimental setup. However, this method requires a model of the photoinduced carrier dynamics to be known a priori.

Supplementary Note 3: Experimental details of the ultrafast measurements

A. Photoexcitation scheme

In our ultrafast THz experiments, we photoexcite $NiPS_3$ in the spectral region of the spin–orbit-entangled excitons. We use a pump photon energy of 1.55 eV, which lies well below the tail of the charge-transfer (CT) gap. To verify this, in Supplementary Fig. 5 we show the optical absorption of $NiPS_3$ in equilibrium over an extended energy range compared to that of Fig. 1b in the main text. The blue curve is the data from Fig. 1b, and the red curve is determined from ellipsometry data presented in Ref. [6]. This plot of the absorption coefficient demonstrates that our pump photon energy of 1.55 eV is indeed well below the CT gap. From the absorbed pump fluence and photoexcited volume, we estimate an initial density of excitons on the order of 10^{19} cm^{-3}. A fraction

($\sim 10^{-5}$) of these excitons subsequently dissociate, giving rise to itinerant carriers that produce the Drude response (see Supplementary Note 4 for a calculation of the density of the itinerant carriers). Therefore, in the photoinduced state there is a coexistence of excitons (that have not dissociated) and mobile carriers. These two populations are likely to follow independent dynamics and thus there is no contradiction between the time evolution of the Drude response and the exciton coherence lifetime. Moreover, we note that the exciton coherence lifetime is at least 10 ps. This lower bound is set by the linewidth of the exciton resonance in the equilibrium optical absorption spectrum (0.4 meV)[7], which is limited by the experimental resolution and also accounts for possible inhomogeneous broadening.

B. Rise time of the pump-probe traces

Supplementary Fig. 6 shows a representative trace of the pump-induced change in the THz electric field (ΔE) in $NiPS_3$ along with the pump-probe signal of a test sample of high-resistivity silicon (Si). The signal of Si stems from the free-carrier absorption in the THz range that follows above-gap excitation at 1.55 eV. The rise in both curves is identical, indicating that the rise time is limited by the intrinsic time resolution of our THz setup. In this plot, the absorbed fluence is 0.3 mJ/cm^2 for Si and 0.5 mJ/cm^2 for $NiPS_3$, but all fluences showed the same resolution-limited rise time.

Supplementary Note 4: Nature of the Drude response

To obtain more quantitative information about the exciton-driven conducting state in $NiPS_3$, we examine the pump-induced change in the optical conductivity as a function of frequency at the pump-probe delay of the maximum Drude response. Supplementary Figs. 7a and 7b show the real ($\Delta\sigma_1$) and imaginary ($\Delta\sigma_2$) parts, respectively, of

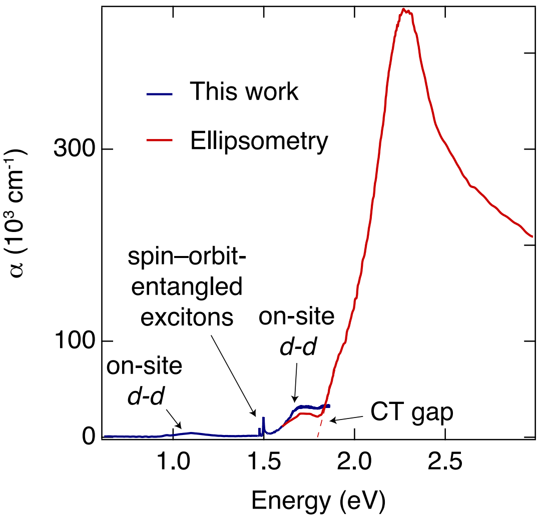

Supplementary Fig. 5 Optical absorption of $NiPS_3$ over an extended energy range. Absorption coefficient (α) in equilibrium. The blue curve is the absorption data shown in Fig. 1b in the main text and the red curve is the absorption coefficient determined from ellipsometry measurements reported in Ref. [6]. The former includes the two broad on-site d-d transitions around 1.1 and 1.7 eV and the narrow spin–orbit-entangled excitons around 1.5 eV, and the latter shows the energy of the charge-transfer (CT) gap. The absorption plotted over this extended energy range highlights the fact that our pump pulse energy of 1.55 eV lies well below the tail of the CT gap.

the change in conductivity at a time delay of 1.6 ps. The conductivity values that we observe are comparable to those reported in previous experiments on other materials in which excitons dissociate[8,9]. Ignoring the magnon part of the spectrum, the two curves can be accurately fit simultaneously to a Drude response using the program RefFIT[10]. As mentioned in the main text, we extracted a plasma frequency of $\omega_p = 4.7$ meV and a total scattering rate of $\gamma = 4$ meV. From these values, we can estimate the carrier mobility to be $\mu \sim 1800$ cm^2/(Vs), relying on the bare electron mass. This value changes to $\mu \sim 1100 - 2300$ cm^2/(Vs) when taking into account the carrier effective mass estimated from electronic structure calculations ($m^* \sim 0.78 - 1.6\, m_e$)[11]. From the values of ω_p

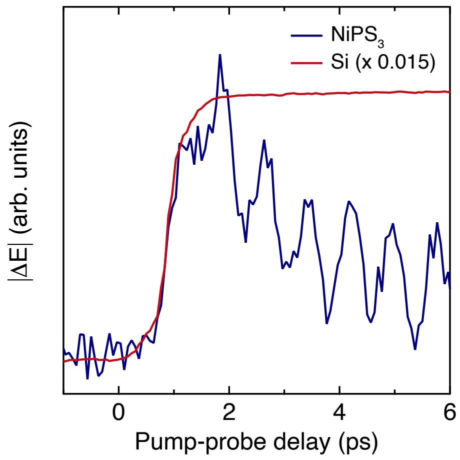

Supplementary Fig. 6 Resolution-limited rise time of the THz transmission experiment. Comparison of the pump-induced change in the THz electric field (ΔE) in $NiPS_3$ and a test sample of high-resistivity silicon. The rise of the pump-induced THz electric field in both cases is identical, indicating that it is limited by the intrinsic time resolution of our setup.

and m^*, we also estimate that the density of itinerant carriers is in the range of 3×10^{14} to 6×10^{14} cm^{-3}. This density can be further increased by increasing the pump fluence. In our ultrafast THz spectroscopy experiments, this is hindered by the constraint that the pump spot size must be larger than the THz probe to ensure a uniform illumination.

Next, we establish that the time evolution of the Drude signal is solely governed by the change in ω_p and not by the variation of γ. This information is encoded in $\Delta\sigma_2$ (Supplementary Fig. 8) since the peak associated with the reactive part of the Drude response provides an estimate of the total scattering rate γ[3, 12]. Supplementary Fig. 8a shows the full spectro-temporal evolution of $\Delta\sigma_2$, and Supplementary Fig. 8b is a top view of Supplementary Fig. 8a zoomed into lower energies corresponding to the Drude contribution. We observe that the peak in the $\Delta\sigma_2$ signal remains constant around 4 meV over time. Another visualization of this feature is offered in Supplementary Fig. 8c,

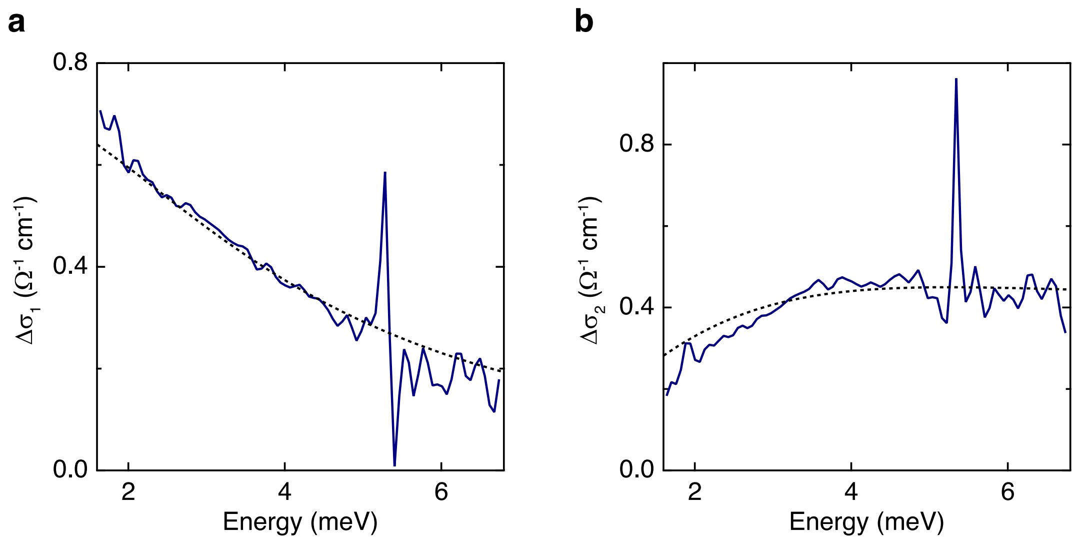

Supplementary Fig. 7 Fit of the photoinduced Drude response. Pump-induced change in the real ($\Delta\sigma_1$, **a**) and imaginary ($\Delta\sigma_2$, **b**) parts of the optical conductivity as a function of frequency at a pump-probe delay of 1.6 ps. The blue curves represent the data and the black dashed lines are fits to a Lorentzian centered at zero frequency.

where the $\Delta\sigma_2$ spectrum is compared at a few representative pump-probe delays. The observation that γ does not vary in time indicates that the mobile carriers produced by exciton dissociation are cold and lie around the band edges[13]. This is in stark contrast to the cooling processes that are still active after 1 ps in materials where high-energy bosons are emitted upon injection of hot carriers[14]. Consequently, the exciton dissociation in $NiPS_3$ results in mobile carriers that do not possess enough excess energy to emit hot optical phonons and high-frequency magnons, which is a crucial aspect in the preservation of the underlying antiferromagnetic order at all time delays (signaled by the persistence of the sharp $q = 0$ magnon mode). Further evidence that the decay of the Drude response is due to a decrease in ω_p is given by the depletion in the spectral weight of $\Delta\sigma_1$ over time (Supplementary Fig. 14), as this quantity is directly proportional to ω_p^2 by the optical sum rule.

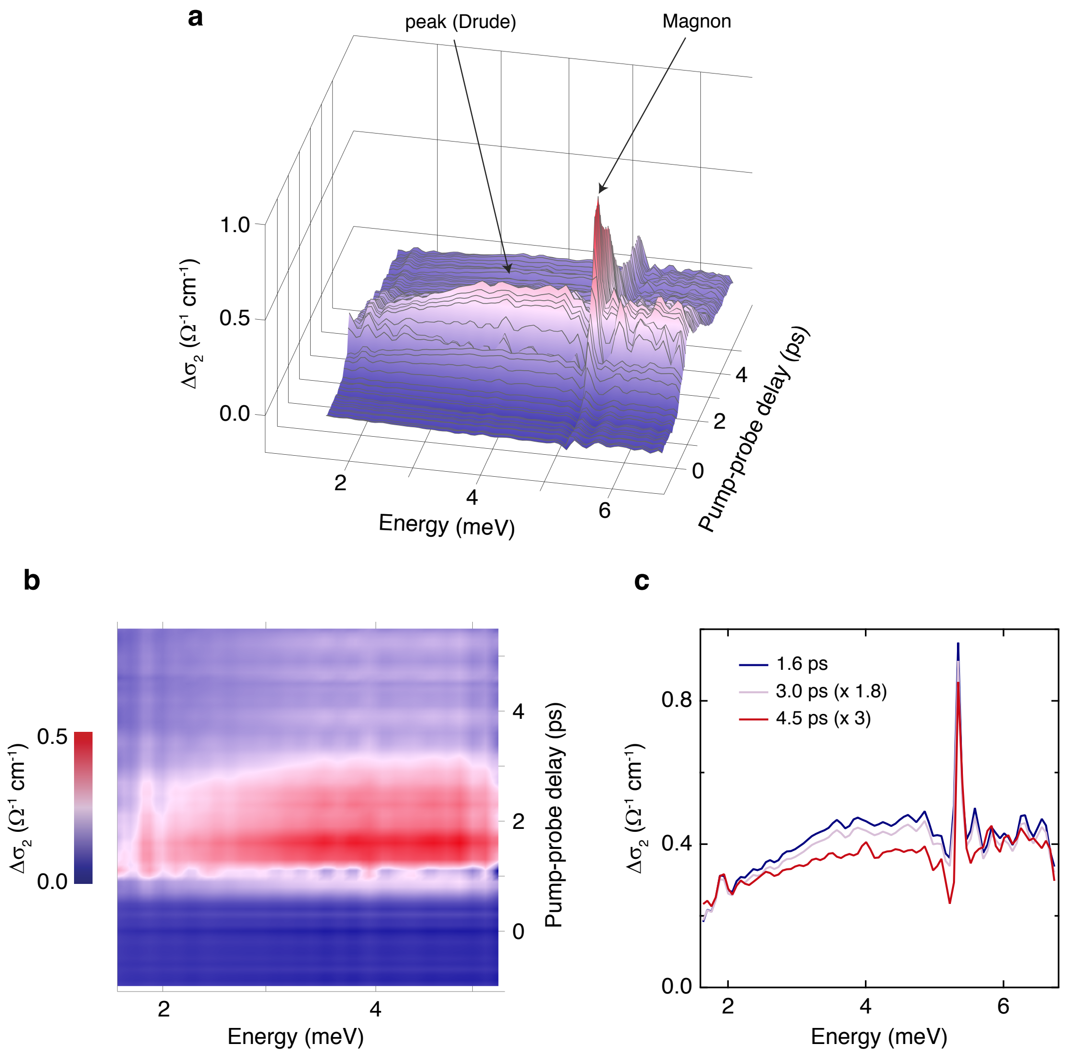

Supplementary Fig. 8 Time independence of the Drude scattering rate. **a**, Spectro-temporal evolution of the pump-induced change in the imaginary part of the optical conductivity ($\Delta\sigma_2$). The temperature is 20 K and the absorbed pump fluence is 1.3 mJ/cm^2. The two features present are a broad peak around 4 meV associated with the Drude response and a Lorentzian lineshape around the magnon energy. **b**, Top view of **a** showing only the Drude contribution. **c**, Spectral dependence of $\Delta\sigma_2$ at a few representative pump-probe delay times. We see that the peak of the $\Delta\sigma_2$ signal does not vary over time, indicating a constant scattering rate.

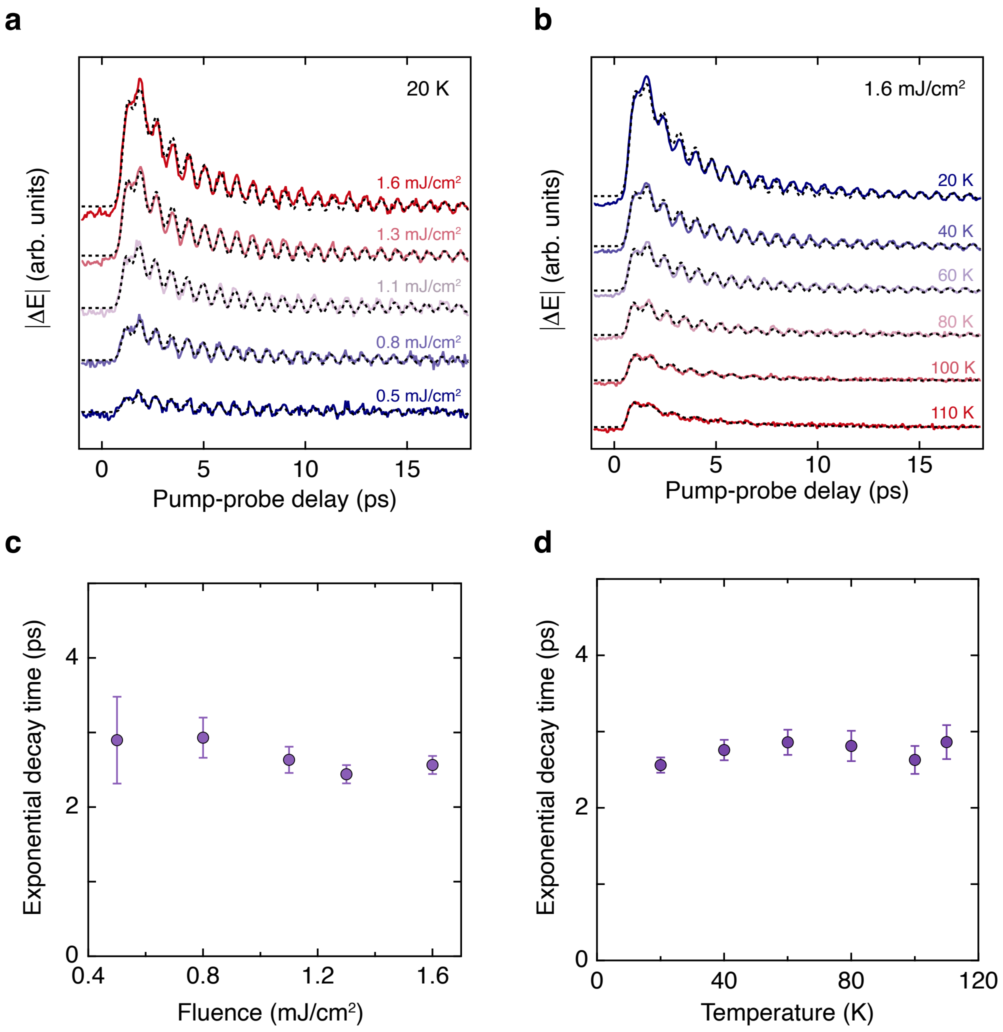

Supplementary Fig. 9 Fluence and temperature independence of the Drude decay time. **a**,**b**, Temporal evolution of the pump-induced change in the THz electric field (ΔE) of the spectrally-integrated measurement as a function of absorbed pump fluence at 20 K (**a**, same as Fig. 2d in the main text) and as a function of temperature at an absorbed pump fluence of 1.6 mJ/cm^2 (**b**). **c**,**d**, Dependence of the exponential decay time on the absorbed pump fluence (**c**) and on temperature (**d**) extracted from the fits to the data in **a** and **b**, respectively. There is almost no variation in the decay time in either case, demonstrating that the itinerant carrier recombination dynamics is dominated by their localization in deep traps and that carriers are not subsequently thermally activated. The error bars in **c** and **d** represent the 95% confidence interval of the fits.

For these reasons, we can relate the decay of the Drude conductivity to the recombination dynamics of the mobile carriers. To unravel which recombination mechanisms contribute to the decay, we measure the ultrafast THz transmission as a function of absorbed fluence (Supplementary Fig. 9a, same as Fig. 2d in the main text) and temperature (Supplementary Fig. 9b). We observe that all the temporal traces can be fit with only one exponential function, indicating the presence of a single recombination pathway for the mobile carriers. The fluence dependence of the exponential decay time is shown in Supplementary Fig. 9c. The decay time is nearly independent of fluence, which suggests that the carrier recombination dynamics is governed by their trapping or self-trapping at deep impurity centers or defects as described by Shockley-Read-Hall (SRH) theory[15,16]. The general equation governing the lifetime τ of free carriers in semiconductors is $\frac{1}{\tau} = A + Bn + Cn^2$, where n is the carrier density and A, B, and C are the coefficients for nonradiative recombination (SRH), radiative recombination, and Auger recombination, respectively[17]. Here, the first term is responsible for the observed nearly fluence-independent decay time. In Supplementary Fig. 9d, we show the temperature dependence of the exponential decay time. The decay time is also constant with temperature, confirming that the trapped levels are deep and no thermal activation of carriers occurs after their localization[18]. Therefore, the depletion of the THz spectral weight is accompanied by its transfer to another spectral region outside of our measured range, most likely in the mid- or near-infrared (i.e. where impurity centers and small polarons absorb light)[19].

We also verified that the observed Drude signal stems from the transport of quasi-free carriers and it is not due to the band-like motion of large polaronic carriers[20,21]. In order for a large polaron to form in a deformable medium, the long-range Coulomb potential $V^{LR}(\mathbf{r})$ between an excess carrier and the ionic lattice must be large. This

potential can be expressed as

$$V^{LR}(\mathbf{r}) = -\left[\frac{1}{\epsilon_r(\infty)} - \frac{1}{\epsilon_r(0)}\right]\frac{e^2}{|\mathbf{r}|\epsilon_0}, \tag{8}$$

where $\mathbf{r}$ is the vector between an electron and an ionic site, e is the electron charge, ϵ_0 is the vacuum permittivity, and $\epsilon_r(0)$ and $\epsilon_r(\infty)$ are the static and high-frequency dielectric constants, respectively. The form of the expression ensures that the fast electronic contribution to the polarizability is canceled and only the nuclear contribution is taken into account. As established in polaron theory[22], large polaron formation is possible only when $\epsilon_r(0)$ is at least twice the value of $\epsilon_r(\infty)$. In $NiPS_3$, our static THz data show that $\epsilon_r(0)$ = 7.9. In contrast, $\epsilon_r(\infty)$ can be extracted from optical spectroscopy data in a frequency range that is above the relevant longitudinal optical phonon energies but below the energy of the lowest interband transitions. A reasonable choice of the photon energy in $NiPS_3$ is in the range $0.3 - 1$ eV. At these energies $\epsilon_r(\infty)$ = $9.3 - 10$[6,23], i.e. a value that is even larger than $\epsilon_r(0)$. For this reason, we can rule out a large polaron origin for the coherent Drude response detected in our ultrafast THz transmission experiment and conclude that the carriers involved in the itinerant transport have a quasi-free character. This indicates that the effective mass that is relevant to estimate the carrier mobility is the one extracted from electronic structure calculations that do not account for electron-phonon coupling[11]. Indeed, no mass enhancement due to a Fröhlich-type electron-phonon coupling is expected for $NiPS_3$. These arguments confirm that the carrier mobility lies in the 10^3 cm^2/(Vs) range, i.e. well above the typical carrier mobilities $>$1 cm^2/(Vs) expected from the large polaron scenario (which would require an unreasonable $m^* > 1000 m_e$).

Finally, we remark that the existence of the itinerant conductivity, caused by the dissociation of the photogenerated excitons, is not associated with those excitons having a

coupling to magnetic degrees of freedom. Indeed, bare excitons (regular electron-hole bound states) that dissociate through exciton-exciton interactions or pump-induced exciton photoionization can give rise to photoconductivity as has been found in a variety of band semiconductors[24–28]. However, the Drude signal in $NiPS_3$ becomes stronger with decreasing temperature below T_N (Supplementary Fig. 9b). This is due to the increased exciton optical absorption (i.e. oscillator strength) as the temperature is lowered below T_N. Overall, our main result of the coexistence of the Drude response and the long-wavelength antiferromagnetic magnon is observed only at temperatures below T_N.

Supplementary Note 5: Mechanism of the coherent magnon generation

A. Pump polarization dependence

To establish that the coherent magnon is launched via the photogenerated spin–orbit-entangled excitons, we rule out other conventional mechanisms that are involved in coherent magnon generation in solids. This is achieved through a detailed pump polarization dependence. Supplementary Fig. 10a shows the emitted THz electric field (E) for different linear polarization directions of the pump pulse. There is no change in the phase of the magnon oscillations, which rules out the inverse Cotton-Mouton effect as the generation mechanism[29]. Supplementary Fig. 10b compares the magnon oscillations when the pump pulse is right and left circularly polarized. The same oscillation phase is again observed, this time ruling out the inverse Faraday effect[30].

B. Pump fluence dependence of the magnon energy

In this section, we demonstrate that the dependence of the magnon energy on the absorbed pump laser fluence (shown in Figs. 3e,f in the main text) is non-thermal. Sup-

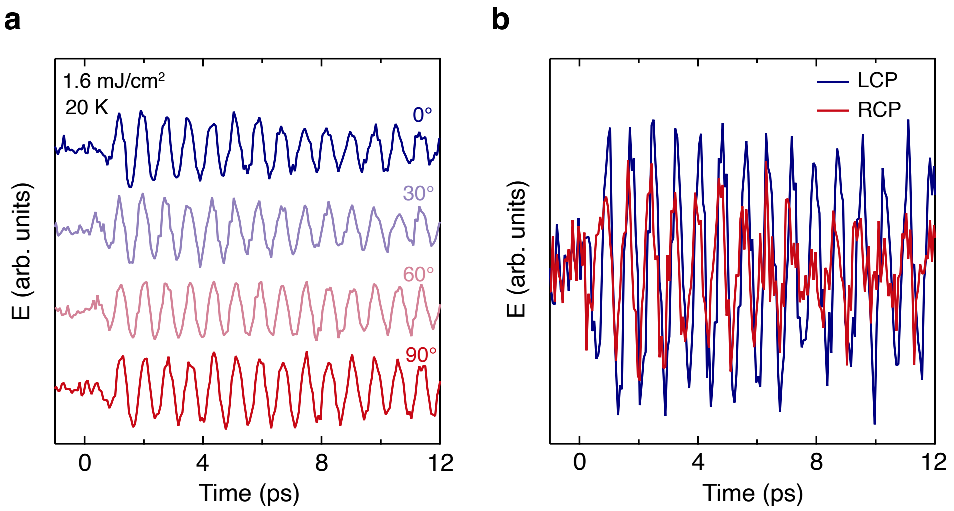

Supplementary Fig. 10 Pump polarization dependence for NiPS$_3$. a, Dependence of the emitted THz electric field (E) on various linear polarization directions of the pump beam. The temperature is 20 K and the absorbed fluence is 1.6 mJ/cm^2. There is no change in phase of the magnon oscillations, ruling out the inverse Cotton-Mouton effect as the mechanism responsible for launching the coherent magnon. **b**, Dependence of the THz emission signal on left (blue) and right (red) circularly polarized light. Again, the oscillation phase remains unchanged, this time indicating that the magnon excitation is not due to the inverse Faraday effect.

plementary Fig. 11 presents the data from Fig. 3f at 110 K (red points) along with the change in energy that would occur thermally from the increase in the lattice temperature due to the pump laser at each fluence (violet points). The change in temperature is calculated from the heat deposited into the sample by the pump at a given fluence and the heat capacity of NiPS$_3$ (Supplementary Fig. 2). Then this calculated temperature change is added to the starting temperature to obtain the effective temperature (x axis of Supplementary Fig. 11) of the sample during the measurement at a particular fluence. The data point at 110 K corresponds to the magnon energy in equilibrium (no pump beam) and is obtained by interpolating the order-parameter-like dependence of the energy as a function of temperature in equilibrium (Fig. 3d in the main text, violet points). Thus,

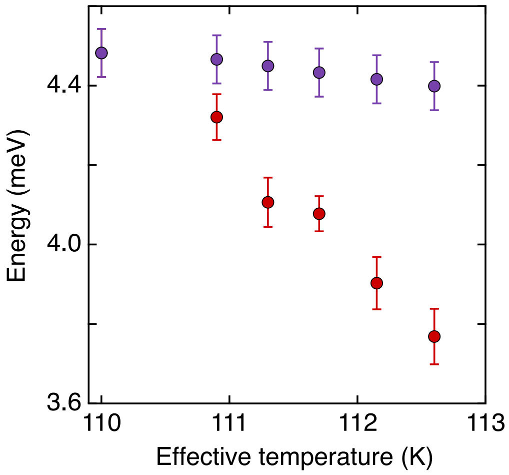

Supplementary Fig. 11 Non-thermal nature of the fluence dependence of the magnon energy. Dependence of the magnon energy on the effective temperature of the sample. The red points are extracted from the THz emission measurements upon photoexcitation at 110 K (same as those shown in Fig. 3f in the main text). The violet points represent the thermal change in the magnon energy and are calculated from the heat deposited in the sample for each absorbed pump fluence and the heat capacity data. The violet data point at 110 K corresponds to the magnon energy in equilibrium at this temperature. This confirms that our photoexcitation mechanism is non-thermal in nature. The error bars are similarly defined as in Figs. 3d,f in the main text.

from Supplementary Fig. 11 we can see that the decrease in energy observed in the THz emission experiment (red points) is substantially larger than what would be detected if the action of the pump was purely thermal in nature (violet points). Further evidence that the mechanism responsible for launching the coherent magnon is non-thermal is that the magnon oscillations begin during the rise of the Drude, as discussed in the main text.

C. THz transmission and THz emission comparison

In this section, we verify that the oscillations observed in the THz transmission experiment (Fig. 2 in the main text) are the same magnon oscillations detected with THz

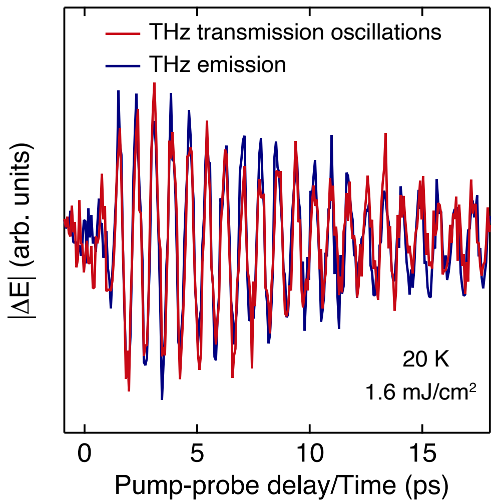

Supplementary Fig. 12 Comparison of magnon oscillations in the THz transmission and emission experiments. Oscillations from the THz transmission experiment (red) plotted with the THz emission signal (blue), both at 20 K and with an absorbed fluence of 1.6 mJ/cm^2. The traces are nearly identical, confirming the magnon origin of the THz transmission oscillations.

emission (Fig. 3 in the main text). Supplementary Fig. 12 displays the THz transmission oscillations (the 1.6 mJ/cm^2 curve in Fig. 2d in the main text after subtracting the exponential background) along with the results of a THz emission measurement with the same experimental parameters. The two traces are nearly identical, confirming the magnon origin of the coherent oscillations present in the THz transmission data.

Supplementary Note 6: Significance of our non-equilibrium results and comparison to band semiconductors

In this section, we describe how the current results on $NiPS_3$ represent an anomalous phenomenon that has no counterpart in the physics of photoexcited band semiconductors.

In pristine semiconductors devoid of strong electronic correlations, the charge gap

arises because of band theory arguments. Photoexciting electron-hole pairs above this band gap (up to carrier densities of 10^{19}-10^{21} cm^{-3}) typically results in the appearance of a Drude response in the THz or far-infrared range due to free-carrier (intraband) absorption[4]. Therefore, during the time that precedes complete electron-hole recombination (varying from several picoseconds to nanoseconds depending on the system), the initially undoped band semiconductor transiently develops metallic conductivity, signaled by the appearance of a Drude response[31]. This effect has been widely studied in semiconductor physics to estimate the carrier transport and mobility in materials of optoelectronic interest[4], to realize switchable optical components[32,33], or to clarify the optical nonlinearities affecting a semiconductor's exciton resonances[34].

It is well known that the above-gap photoexcitation process described above also often results in the generation of phonons that coherently evolve as a function of time[35]. These phonons can be optical or acoustic, and they can be generated at $q = 0$ or at finite momentum[36,37]. In band semiconductors, coherent phonons are usually triggered via a non-thermal deformation potential coupling (i.e. the so-called displacive excitation in the case of optical phonons)[38,39], but also other mechanisms (e.g., thermoelasticity, piezoelectric coupling, etc.) can play a role[40]. These coherent phonons coexist and interact with the electron-hole plasma created by the pump pulse. The real (frequency) and imaginary (decay rate) parts of the self-energy are therefore profoundly affected by this electron-phonon interaction[31,37,41]. Nevertheless, the phonons still represent well-defined collective modes of the lattice at all momenta because the crystal has neither undergone a structural phase transition nor has been melted by the photoexcitation.

The scenario emerging in our experiments on $NiPS_3$ is radically different and cannot be viewed as a simple extension of the previous case to coherent magnons. In a Mott insulator, the charge gap stems from strong electronic correlations and it is not a result of

band theory[42]. Antiferromagnetic order typically develops at low temperature to reduce the ground-state energy of the system, with magnons emerging as well-defined collective modes. When above-gap light excitation photodopes a Mott insulator, the presence of the itinerant carriers is always accompanied by the melting of the long-range antiferromagnetic order[43–45]. At short timescales, this melting can proceed through different mechanisms, such as the collapse of the magnetic moments, the quench of the exchange interaction, or the direct injection of energy from the hot photocarriers into the spin system[46]. Regardless of the detailed pathway, the long-wavelength magnon—which signals the presence of long-range antiferromagnetic order—always collapses by broadening and losing intensity. This is also observed in the insulator-to-metal transition realized by chemical doping a Mott insulator[47]. Consequently, only short-range magnetic correlations survive and are reflected in the persistence of paramagnons at short-wavelength (finite q) and high energy[43,45]. For these reasons, the coexistence of a Drude response and a very sharp $q = 0$ magnon in $NiPS_3$ is an unexpected and hitherto-unobserved finding, markedly different from the melting dynamics reported thus far in photoexcited Mott insulators.

Supplementary Note 7: Homogeneity of the photoinduced state

In the main text, we indicate that our results preclude an interpretation of the antiferromagnetic conducting state as a phase-separated state composed of metallic patches embedded in an antiferromagnetic insulator. Below we explain in detail the features of our data that rule out this alternative scenario.

First, we consider the lineshape of the magnon mode. The sharpness of the magnon feature displayed in Fig. 2b in the main text and its lack of any noticeable broadening indicates that the magnon remains a well-defined mode after the arrival of the pump

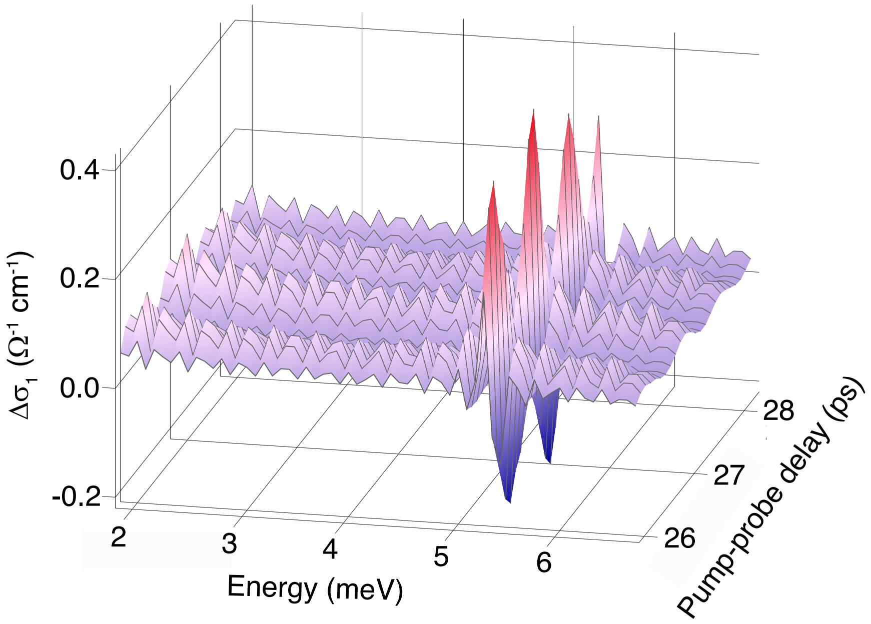

Supplementary Fig. 13 Spectro-temporal evolution of the conductivity at later delay times. Spectro-temporal evolution of the pump-induced change in the real part of the optical conductivity ($\Delta\sigma_1$) for pump-probe delay times ranging from 26 to 28 ps. The temperature is 20 K and the absorbed pump fluence is 1.3 mJ/cm^2. In contrast to Fig. 2b in the main text, at these later delay times the Drude response has disappeared and only the first-derivative-like shape around the magnon energy remains. The coherent magnon oscillations as a function of time are still present as well.

pulse and it does not evolve into a paramagnon[47]. If we suppose that the pump creates paramagnetic metallic patches, these regions would act like impurities in the insulating antiferromagnetic system, thereby disrupting the long-range antiferromagnetic order and decreasing its correlation length. Consequently, the presence of the metallic puddles would lead to a broadening of the long-wavelength ($q = 0$) magnon that would increase with fluence as the volume of the metallic regions increases[47,48]. However, the damping of our magnon oscillations does not show any noticeable change as a function of fluence. Moreover, the magnon lineshape retains the same sharp width at all pump-probe time delays both while the mobile carriers are present and after they disappear

once the Drude decays (after ~ 15 ps). The former is shown in Fig. 2b in the main text and the latter is presented in Supplementary Fig. 13. From Supplementary Fig. 13, we see that the 2D map of $\Delta\sigma_1$ contains only the sharp, first-derivative-like shape of the magnon without the Drude response, and the magnon is oscillating coherently as a function of pump-probe delay. The same behavior was observed up to 80 ps after the pump pulse arrival. To better visualize the spectra before and after the Drude decays, in Supplementary Fig. 14 we plot $\Delta\sigma_1$ as a function of frequency at several representative pump-probe delay times. Comparing the traces, we see that the magnon retains its narrow lineshape and therefore is not affected by the presence or absence of the mobile carriers. Thus, our observations are inconsistent with the scenario of phase-separated, paramagnetic metallic patches.

Next, we examine the fluence dependence of the magnon oscillation amplitude. Since the coherent magnon is generated by the pump excitation due to the coupling of the spin–orbit-entangled excitons with the magnetic order, its amplitude at times < 1 ps should be linearly proportional to the number of excitons created. Afterwards, exciton dissociation into free carriers through mutual collisions produces a Drude conductivity in the system. Due to the time resolution of our ultrafast THz experiment (see Supplementary Note 3B), the magnon amplitude can be evaluated only by fitting the oscillation that emerges on top of the Drude response, i.e. after the exciton dissociation process is complete (> 1 ps). If the dissociated excitons formed separate, paramagnetic metallic patches, then the size of the insulating antiferromagnetic region would be reduced and hence the magnon precession amplitude would become smaller than that initially generated by the pump. With increasing fluence, the initial magnon amplitude would rise linearly but the volume of metallic patches would increase quadratically (as determined by the quadratic dependence of the exponential amplitude in Fig. 2f in the main text),

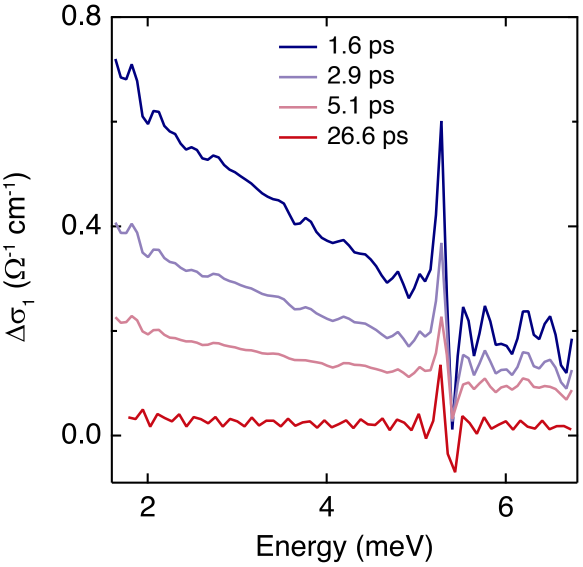

Supplementary Fig. 14 Comparison of the conductivity over time. Pump-induced change in the real part of the optical conductivity ($\Delta\sigma_1$) as a function of frequency at several representative pump-probe delay times: 1.6 ps (at the peak of the Drude response), 2.9 and 5.1 ps (during the decay of the Drude), and 26.6 ps (after the Drude has disappeared). These traces are cuts taken from the 2D maps shown in Fig. 2b in the main text (1.6, 2.9, and 5.1 ps) and Supplementary Fig. 13 (26.6 ps). The first-derivative-like shape around the magnon energy remains sharp at all times, indicating that the mobile carriers do not affect the width of the magnon lineshape.

leading to a reduction in the magnon amplitude that scales quadratically as a function of fluence. Hence, the observed magnon amplitude (>1 ps) would show a sublinear trend with fluence. In contrast, as presented in Fig. 2e in the main text, the amplitude of the magnon evaluated after 1 ps still scales linearly with fluence, as if no changes occur when the excitons dissociate compared to when the excitons are initially generated. This feature is expected when the itinerant carriers coexist with the underlying long-range antiferromagnetism and do not interfere with the long-wavelength precession of the localized spins.

Thus, combining these aspects of our data, an interpretation involving phase sepa-

ration of paramagnetic metallic patches embedded in the antiferromagnetic insulating state can be readily ruled out, and we conclude that our data indeed demonstrates a coexistence of a finite itinerant conductivity and antiferromagnetism.

Finally, the fact that our photoexcited state is homogeneous in nature allows us to estimate that the itinerant charge carriers are separated by a distance of $\sim$120-150 nm. Here we argue that this distance is much smaller than the magnetic correlation length in the excited state, the latter being similar to the static magnetic correlation length. Indeed, we note that the magnon mode we observe is the lowest-energy (pseudo-Goldstone) mode at $q = 0$, a robust fingerprint of the long-range antiferromagnetic order. In the transient state, there is no broadening of this magnon mode, thus confirming that the static long-range magnetic order is preserved and is not modified significantly by the presence of the itinerant carriers. Ideally, the static correlation length can be extracted from the linewidth of the magnetic Bragg peak in diffraction experiments. However, for single crystals of $NiPS_3$ there are currently no elastic neutron/x-ray scattering data from which this static correlation length can be estimated. In practice, the static magnetic correlation length is limited by the size of the magnetic domains in the crystal. In $M$$PS_3$ materials (where M is a transition metal), these domains have a size on the order of a few μm (i.e. $\gg$ 120-150 nm)[49].

Supplementary Note 8: $MnPS_3$ and $FePS_3$

To better understand the behavior observed in $NiPS_3$, it is useful to compare the results on $NiPS_3$ presented in the main text to those of closely related van der Waals antiferromagnets. In this respect, we focus on the compounds $MnPS_3$ and $FePS_3$, whose Néel temperatures are ~ 78 K and ~ 120 K, respectively. Inelastic neutron scattering measurements show that there are no magnon resonances in our observable energy range in

either material (the lowest-energy magnon resonances were found to be $\sim$0.5 meV[50] and $\sim$15 meV[51] for $MnPS_3$ and $FePS_3$, respectively). We confirm the absence of any magnon modes by measuring the equilibrium THz absorption as a function of temperature.

We further note that neither $MnPS_3$ nor $FePS_3$ shows any pump-probe signal (Drude absorption) when we excite each system with an ultrashort near-infrared pump pulse, in contrast to $NiPS_3$. The lack of a non-equilibrium response can be understood by examining optical absorption data for these compounds. Unlike $NiPS_3$, which exhibits sharp spin–orbit-entangled excitons in the vicinity of our pump photon energy[7], $MnPS_3$ and $FePS_3$ are purely transparent at 1.55 eV, with on-site d-d transitions lying elsewhere and the optical charge-transfer gap at higher energies ($E_{\text{gap}} \sim 2.9$ eV for $MnPS_3$ and ~ 1.8 eV for $FePS_3$ at low temperature)[52–57].

Supplementary Note 9: Theoretical calculations

A. Model Hamiltonian for $NiPS_3$

As mentioned in the main text, the crystal structure of $NiPS_3$ consists of two dimensional (2D) layers of Ni atoms arranged in a honeycomb lattice in the ab-plane that are coupled in the c direction by the van der Waals interaction. Each individual layer is invariant under a C_3 rotation but the stacking pattern breaks this symmetry and induces an anisotropy along the a-axis.

In the resulting effective spin model, the Ni sites have spin-1 and no orbital degeneracy. Assuming the interlayer coupling is small, for each layer the effective spin Hamiltonian consists of XXZ terms up to third-nearest neighbors and single-ion anisotropy

along the a-axis and along the c-axis[58]:

$$\begin{aligned}H_{spin} =& J_1 \sum_{\langle i,j \rangle} (S_{i,x}S_{j,x} + S_{i,y}S_{j,y} + \alpha S_{i,z}S_{j,z}) \\ &+ J_2 \sum_{\langle\langle i,j \rangle\rangle} (S_{i,x}S_{j,x} + S_{i,y}S_{j,y} + \alpha S_{i,z}S_{j,z}) \\ &+ J_3 \sum_{\langle\langle\langle i,j \rangle\rangle\rangle} (S_{i,x}S_{j,x} + S_{i,y}S_{j,y} + \alpha S_{i,z}S_{j,z}) \\ &+ D_x \sum_i S_{i,x}^2 + D_z \sum_i S_{i,z}^2,\end{aligned} \tag{9}$$

where J_1, J_2, J_3 are the nearest neighbor, second-nearest neighbor, and third-nearest neighbor coupling, α is the anisotropic spin-spin interaction parameter, and $D_{x,z}$ are the single-ion anisotropic coefficients. A small D_x breaks $U(1)$ down to Z_2, resulting in an Ising transition at finite temperature. In the single layer limit, the $U(1)$ symmetry is restored so there will be a Kosterlitz-Thouless (KT) transition at finite temperature. We ignore interlayer couplings here since they are much smaller than the intralayer interactions.

B. Classical ground state

When the spin operators are treated as classical variables, we can diagonalize the spin Hamiltonian in momentum space. Note that S_x, S_y, and S_z are decoupled and there are two atoms (A and B) in a honeycomb unit cell. We only need to diagonalize three 2×2 matrices. We denote each block by $h_x(\vec{k})$, $h_y(\vec{k})$, and $h_z(\vec{k})$:

$$h_x(\vec{k}) = (S_{x,A}(\vec{k})\ S_{x,B}(\vec{k})) \begin{pmatrix} Q_2(k) + D_x & Q_1(k) + Q_3(k) \\ Q_1^*(k) + Q_3^*(k) & Q_2(k) + D_x \end{pmatrix} \begin{pmatrix} S_{x,A}(-\vec{k}) \\ S_{x,B}(-\vec{k}) \end{pmatrix} \tag{10}$$

$$h_y(\vec{k}) = (S_{y,A}(\vec{k})\ S_{y,B}(\vec{k})) \begin{pmatrix} Q_2(k) & Q_1(k)+Q_3(k) \\ Q_1^*(k)+Q_3^*(k) & Q_2(k) \end{pmatrix} \begin{pmatrix} S_{y,A}(-\vec{k}) \\ S_{y,B}(-\vec{k}) \end{pmatrix} \tag{11}$$

$$h_z(\vec{k}) = (S_{z,A}(\vec{k})\ S_{z,B}(\vec{k})) \begin{pmatrix} \alpha Q_2(k)+D_z & \alpha(Q_1(k)+Q_3(k)) \\ \alpha(Q_1^*(k)+Q_3^*(k)) & \alpha Q_2(k)+D_z \end{pmatrix} \begin{pmatrix} S_{z,A}(-\vec{k}) \\ S_{z,B}(-\vec{k}) \end{pmatrix}, \tag{12}$$

where $Q_1(k) = J_1(e^{ik_y}+e^{-i(\sqrt{3}k_x/2+k_y/2)}+e^{-i(-\sqrt{3}k_x/2+k_y/2)})$, $Q_2(k) = 2J_2[\cos(\sqrt{3}k_x)+\cos(\sqrt{3}k_x/2+3k_y/2)+\cos(-\sqrt{3}k_x/2+3k_y/2)]$, and $Q_3 = J_3(e^{-2ik_y}+e^{i(\sqrt{3}k_x+k_y)}+e^{i(-\sqrt{3}k_x+k_y)})$. Here, we set the distance between the nearest A and B atoms to be 1 and the honeycomb a-axis to be aligned with the x-axis.

Since $D_x < 0$ and all three matrices have a similar k dependence, it follows that the lowest-energy state will be fully polarized along the a-axis. For the given parameters, one can show that the band bottoms are at $(0, \pm 2\pi/3)$ and all the points are related by a C_3 rotation. The eigenvector at $k_+ = (0, 2\pi/3)$ for the lower band is $(e^{i\pi/3}, 1)^T$ with some normalization factor. Performing an inverse Fourier transformation, we obtain $S_{A,x}(\vec{r}) = 2S\cos(\frac{2\pi}{3}(y+\frac{1}{2}))$ and $S_{B,x}(\vec{r}) = 2S\cos(\frac{2\pi}{3}y)$. This is the zigzag order.

C. Symmetries of the zigzag order

The magnetic unit cell is twice as large as the original honeycomb unit cell and it contains four atoms (see Supplementary Fig. 15). Apart from the lattice translation symmetry, we have:

- Magnetic translation $T_{1/2}\mathcal{T}$: translation along the $A_1 - A_2$ direction followed by time reversal $\mathcal{T}$;

- Inversion $\mathcal{I}$: inversion centers at the midpoint of the $A_1 - B_1$ bond and the $A_2 - B_2$ bond;
- $C_2\mathcal{T}$: π rotation along the $A_1 - B_2$ axis followed by time reversal.

All other symmetries can be generated by the above transformations. In order to facilitate the discussion of degeneracy in the magnon spectrum, it is useful to consider the symmetry actions in terms of gliding planes and screw axes. There is a screw axis 2_1 along the y-axis and a gliding plane a lying in the xz-plane followed by time reversal, denoted by $a\mathcal{T}$. Furthermore, $C_2\mathcal{T}$ and $\mathcal{I}$ can be combined, which yields a mirror symmetry, labeled σ (see Supplementary Fig. 15).

D. Magnon dispersion

We perform the Holstein-Primakoff (HP) transformation to find the magnon dispersion. The general form of the HP transformation is:

$$\begin{aligned} S_+ &= \hbar\sqrt{2S - a^\dagger a} a \\ S_- &= \hbar a^\dagger \sqrt{2S - a^\dagger a} \\ S_z &= \hbar(S - a^\dagger a). \end{aligned} \tag{13}$$

In the zigzag order, there are four atoms per magnetic unit cell labeled A_1, A_2, B_1, and B_2 (see Supplementary Fig. 15). Let the spins on the A_1 and B_1 sites point in the positive x-direction and the spins on the A_2 and B_2 sites point in the negative x-direction, and we change the basis for the HP transformation. We ignore the higher order terms and

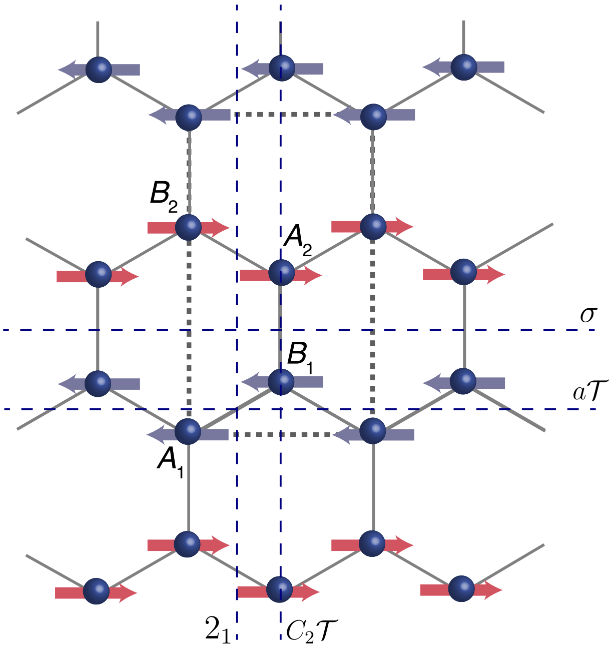

Supplementary Fig. 15 Magnetic unit cell of NiPS$_3$. The thick gray dashed rectangle denotes the magnetic unit cell, which consists of four Ni atoms labeled A_1, A_2, B_1, and B_2. The blue spheres denote Ni atoms and the red and blue arrows represent the spins, showing the zigzag antiferromagnetic order. The thin blue dashed lines represent the symmetries σ, $a\mathcal{T}$, 2_1, and $C_2\mathcal{T}$ of the zigzag order as discussed in the text.

set $\hbar = 1$. Then we have

$$\begin{aligned}
&S_{A_1,i,+} \equiv S_{A_1,i,y} + iS_{A_1,i,z} \approx \sqrt{2S} b_{A_1,i} \\
&S_{A_1,i,-} \equiv S_{A_1,i,y} - iS_{A_1,i,z} \approx \sqrt{2S} b^\dagger_{A_1,i} \\
&S_{A_1,i,x} = S - b^\dagger_{A_1,i} b_{A_1,i} \\
&S_{B_1,i,+} \equiv S_{B_1,i,y} + iS_{B_1,i,z} \approx \sqrt{2S} b_{B_1,i} \\
&S_{B_1,i,-} \equiv S_{B_1,i,y} - iS_{B_1,i,z} \approx \sqrt{2S} b^\dagger_{B_1,i} \\
&S_{B_1,i,x} = S - b^\dagger_{B_1,i} b_{B_1,i} \\
&S_{A_2,i,+} \equiv S_{A_2,i,y} + iS_{A_2,i,z} \approx \sqrt{2S} b^\dagger_{A_2,i} \\
&S_{A_2,i,-} \equiv S_{A_2,i,y} - iS_{A_2,i,z} \approx \sqrt{2S} b_{A_2,i} \\
&S_{A_2,i,x} = -S + b^\dagger_{A_2,i} b_{A_2,i} \\
&S_{B_2,i,+} \equiv S_{B_2,i,y} + iS_{B_2,i,z} \approx \sqrt{2S} b^\dagger_{B_2,i} \\
&S_{B_2,i,-} \equiv S_{B_2,i,y} - iS_{B_2,i,z} \approx \sqrt{2S} b_{B_2,i} \\
&S_{B_2,i,x} = -S + b^\dagger_{B_2,i} b_{B_2,i}.
\end{aligned} \tag{14}$$

Plugging in the HP transformation and taking the limit $\langle b^\dagger b\rangle \ll S$, we can ignore the higher order terms and only keep the boson bilinear terms. Then we obtain

$$\begin{aligned}
H_{Spin} &= (-2J_1+4J_2+6J_3-2D_x+D_z+2J_2P_2(k)(1+\alpha))S\sum_a b^\dagger_{a,k}b_{a,k} \\
&+ J_1S(1-\alpha)P_1^*(k)(b_{A_1,k}b_{B_1,-k}+b_{A_2,k}b_{B_2,-k}) + J_1S(1+\alpha)P_1(k)(b^\dagger_{A_1,k}b_{B_1,k}+b^\dagger_{A_2,k}b_{B_2,k}) + h.c. \\
&+ (J_2S(1-\alpha)P_2(k) - D_zS/2)(b_{A_1,k}b_{A_1,-k}+b_{A_2,k}b_{A_2,-k}+b_{B_1,k}b_{B_1,-k}+b_{B_2,k}b_{B_2,-k}) + h.c. \\
&+ J_2S(1+\alpha)P_3(k)(b_{A_1,k}b_{A_2,-k}+b_{B_1,k}b_{B_2,-k}) + J_2S(1-\alpha)P_3(k)(b^\dagger_{A_1,k}b_{A_2,k}+b^\dagger_{B_1,k}b_{B_2,k}) + h.c. \\
&+ [J_1S(1+\alpha)P_4^*(k)+J_3S(1+\alpha)P_5^*(k)](b_{A_1,k}b_{B_2,-k}+b_{A_2,k}b_{B_1,-k}) \\
&+ [J_1S(1-\alpha)P_4(k)+J_3S(1-\alpha)P_5(k)](b^\dagger_{A_1,k}b_{B_2,k}+b^\dagger_{A_2,k}b_{B_1,k}) + h.c.,
\end{aligned} \tag{15}$$

where $P_1(k) = e^{i(\sqrt{3}k_x/2+k_y/2)} + e^{i(-\sqrt{3}k_x/2+k_y/2)}$, $P_2(k) = 2\cos(\sqrt{3}k_x)$, $P_3(k) = 2\cos(\sqrt{3}k_x/2+3k_y/2) + 2\cos(-\sqrt{3}k_x/2+3k_y/2)$, $P_4(k) = e^{-ik_y}$, and $P_5(k) = e^{2ik_y} + e^{-i(\sqrt{3}k_x+k_y)} + e^{i(\sqrt{3}k_x-k_y)}$, and we ignore the constant term.

For simplicity, let us consider the $\alpha = 1$, $J_1 = J_2 = 0$ case:

$$\begin{aligned}
H_{Spin} &= (6J_3-2D_x+D_z)S\sum_a b^\dagger_{a,k}b_{a,k} \\
&+ (-D_zS/2)(b_{A_1,k}b_{A_1,-k}+b_{A_2,k}b_{A_2,-k}+b_{B_1,k}b_{B_1,-k}+b_{B_2,k}b_{B_2,-k}) + h.c. \\
&+ 2J_3SP_5^*(k)(b_{A_1,k}b_{B_2,-k}+b_{A_2,k}b_{B_1,-k}).
\end{aligned} \tag{16}$$

The Hamiltonian is block-diagonalized into two 4×4 matrices: A_1 and B_2 are coupled and A_2 and B_1 are coupled. The lowest-energy magnon dispersion is

$$\epsilon_k = \sqrt{36J_3^2 - 4J_3^2|P_5(k)|^2 + 12J_3D_z - 4J_3D_z|P_5(k)| - 4D_x(D_z+6J_3) + 4D_x^2} \tag{17}$$

and the magnon gap is given by $\epsilon_0 = \sqrt{-4D_x(D_z+6J_3)+4D_x^2}$.

Let us consider the lowest-energy magnon at $k = 0$, labeled by the bosonic field c_0. Projecting the b fields to the lowest mode, we obtain

$$\begin{aligned} b_{A_1} &= -\sqrt{\frac{J_0 - \epsilon_0}{4\epsilon_0}} c_0^\dagger - \sqrt{\frac{J_0 + \epsilon_0}{4\epsilon_0}} c_0 \\ b_{B_2} &= -b_{A_1} \end{aligned} \tag{18}$$

where $J_0 = 6J_3 - 2D_x + D_z$. In the Heisenberg picture, we have $c_0(t) = c_0 e^{-i\epsilon_0 t}$. The precession of the spins in real space is therefore given by

$$\begin{aligned} S_{A_1,y} &= A\cos(\epsilon_0 t + \phi) \\ S_{A_1,z} &= A'\sin(\epsilon_0 t + \phi) \\ S_{B_2,y} &= -S_{A_1,y} \\ S_{B_2,z} &= -S_{A_1,z}. \end{aligned} \tag{19}$$

This spin precession is illustrated in Supplementary Fig. 16. There is no spatial dependence since we consider the $k = 0$ mode. We note that A, A', and ϕ depend on J_0, ϵ_0, and c_0. The matrices A_2 and B_1 have the same relations as A_1 and B_2. The minus signs in the above equations indicate that the spins on sites A_1 and B_2 precess with opposite chirality, and thus the net magnetization of the sample remains zero.

By diagonalizing the full spin Hamiltonian, we obtain the magnon dispersion. Following the model in Ref. [59], we have $J_1 = -1.9$ meV, $J_2 = 0.1$ meV, $J_3 = 6.9$ meV, $\alpha = 1$, $D_x = -0.3$ meV, and $D_z = 0$ meV. Note that the model has $SO(2)$ symmetry corresponding to a spin rotation along the x-axis. As a result, the magnon dispersion has at least a two-fold degeneracy. However, the $SO(2)$ symmetry is only approximate and we expect the symmetry breaking terms in the spin Hamiltonian to be nonzero in general so that the two-fold degeneracy will be lifted. The dispersion along a certain cut in the magnetic Brillouin zone (see Supplementary Fig. 17) is shown in Supplementary

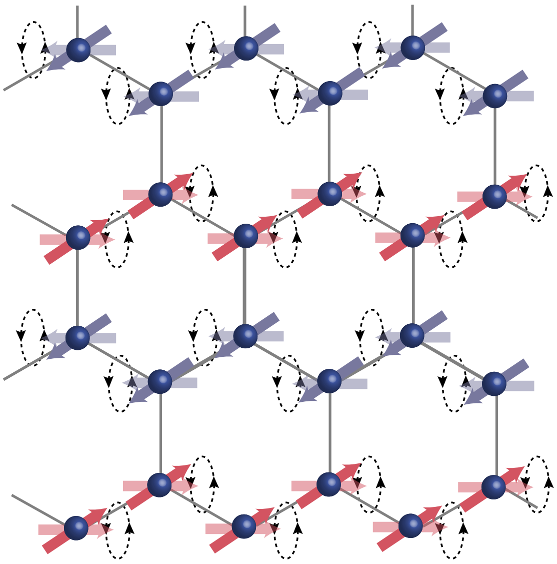

Supplementary Fig. 16 Spin precession of the lowest-energy magnon. Pictorial representation of the spin precession corresponding to the lowest-energy magnon mode. This magnon has zero wavevector, which implies that all the spins precess in phase with each other. The net magnetization remains zero since opposite spins precess with opposite chirality.

Fig. 18. There is a four-fold degeneracy along X-C in the magnetic Brillouin zone. The band minimum is at the zone center with $E_\Gamma \approx 6.81$ meV. There is another magnon at the zone corner with energy $E_C \approx 7.39$ meV, which is close to E_Γ. We remark that the discrepancy between the calculated (6.81 meV) and experimental (5.3 meV) value of the magnon gap at Γ lies in the uncertainties affecting the parameters of the Hamiltonian. Inelastic neutron scattering measurements covering a large energy-momentum window and high energy resolution would allow us to optimize these parameters and refine our estimate.

The four-fold degeneracy can be explained by the space group symmetry of $NiPS_3$. We first examine the two-fold degeneracy of each magnon band. The $SO(2)$ spin ro-

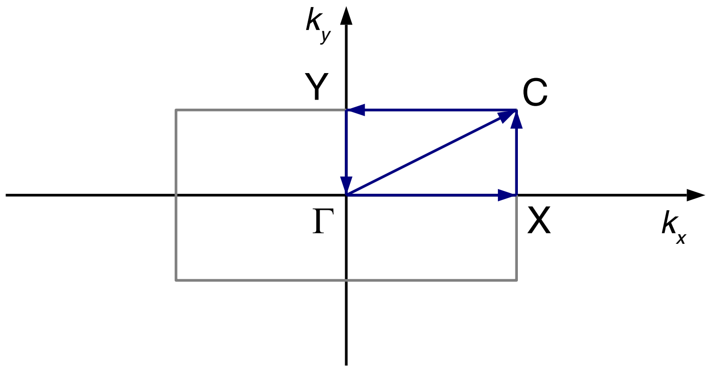

Supplementary Fig. 17 Magnetic Brillouin zone. The gray rectangle denotes the magnetic Brillouin zone. The blue arrows represent cuts along which the magnon dispersion is determined (see Supplementary Figs. 18 and 19).

tation acts on the HP boson as a $U(1)$ rotation, i.e. $b_{A_1,B_1} \to e^{i\theta} b_{A_1,B_1}$ and $b_{A_2,B_2} \to e^{-i\theta} b_{A_2,B_2}$. The Hamiltonian is block-diagonalized into two 4×4 matrices, and the magnetic translation $T_{1/2}\mathcal{T}$ acts as $(b_{A_1}, b_{A_2}, b_{B_1}, b_{B_2}) \to (b_{A_2}, b_{A_1}, b_{B_2}, b_{B_1})$, which ensures that the two blocks in the spin Hamiltonian are identical. The odd and even parity under exchanging A_1, A_2 and B_1, B_2 are essentially degenerate.

Now, we consider the screw axis 2_1 and magnetic translation $T_{1/2}\mathcal{T}$. Note that acting on the lattice, we have $2_1 = \{\left(\begin{smallmatrix} -1 & 0 \\ 0 & 1 \end{smallmatrix}\right)|(\frac{1}{2}, \frac{1}{2})\}$ and $T_{1/2}\mathcal{T} = \{\left(\begin{smallmatrix} 1 & 0 \\ 0 & 1 \end{smallmatrix}\right)|(\frac{1}{2}, \frac{1}{2})\}$. Here, the notation $\{M|\vec{b}\}$ means that a symmetry acts on the coordinates in the 2D plane as $\vec{x} \to M\vec{x} + \vec{b}$, where M is a 2×2 matrix and $\vec{b}$ is a 2D vector. We find that 2_1 and $T_{1/2}\mathcal{T}$ do not commute with each other. Also, we have $T_x(2_1)(T_{1/2}\mathcal{T}) = (T_{1/2}\mathcal{T})(2_1)$, where T_x denotes a translation along the x-axis by one unit cell. At $k_x = \pi$ (the Brillouin zone boundary along the x-axis) $T_x = -1$ so $-(2_1)(T_{1/2}\mathcal{T}) = (T_{1/2}\mathcal{T})(2_1)$. Note that 2_1 does not alter the parity of exchanging A_1A_2 and B_1B_2 so its action is within one 4×4 block of the spin Hamiltonian. There will be an extra degeneracy, i.e. a four-fold

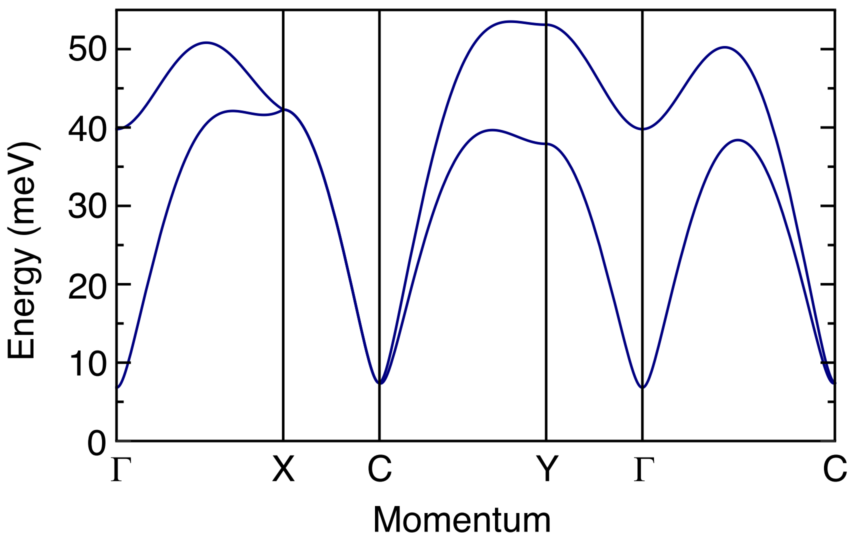

Supplementary Fig. 18 Magnon dispersion with $SO(2)$ symmetry. Magnon dispersion along certain cuts in the magnetic Brillouin zone (see Supplementary Fig. 17) when $SO(2)$ symmetry is preserved. There is a four-fold degeneracy along X-C. The band minimum is at the zone center with $E_\Gamma \approx 6.81$ meV. There is another magnon at the zone corner with energy $E_C \approx 7.39$ meV, which is close to E_Γ.

degeneracy, along the X-C path.

Let us further consider what happens to the magnon spectrum if the $SO(2)$ spin rotation is broken. We use the parameters in Ref. [58]: $J_1 = 1.59$ meV, $J_2 = 2.41$ meV, $J_3 = 4.54$ meV, $\alpha = 0.66$, $D_x = -0.89$ meV, and $D_z = 2.85$ meV. We can see that in general the magnon bands split into four bands but there are two-fold degeneracies along X-C and C-Y (Supplementary Fig. 19). These degeneracies can be explained by the space group symmetry. First, let us consider 2_1 and $C_2\mathcal{T}$. These symmetry actions yield $2_1 = \{(\begin{smallmatrix} -1 & 0 \\ 0 & 1 \end{smallmatrix})|(\frac{1}{2}, \frac{1}{2})\}$ and $C_2\mathcal{T} = \{(\begin{smallmatrix} -1 & 0 \\ 0 & 1 \end{smallmatrix})|(\frac{1}{2}, \frac{1}{2})\}$, and they do not commute with each other. We have $T_x(2_1)(C_2\mathcal{T}) = (C_2\mathcal{T})(2_1)$ so at $k_x = \pi$, we have $-(2_1)(C_2\mathcal{T}) = (C_2\mathcal{T})(2_1)$. This yields at least two-fold degeneracy along X-C in the Brillouin zone. Similar arguments can be carried out for $k_y = \pi$ (the Brillouin zone boundary along the

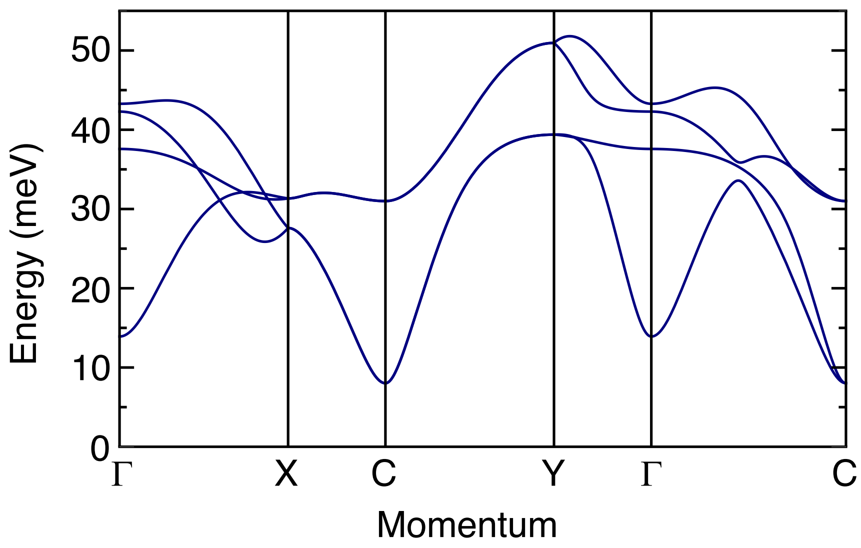

Supplementary Fig. 19 Magnon dispersion without $SO(2)$ symmetry. Magnon dispersion along certain cuts in the magnetic Brillouin zone (see Supplementary Fig. 17) when $SO(2)$ symmetry is broken. In general, the magnon bands split into four bands but there are two-fold degeneracies along X-C and C-Y. These degeneracies can be explained by the space group symmetry of $NiPS_3$.

y-axis) if we consider the symmetries $a\mathcal{T}$ and σ. Note that $a\mathcal{T} = \{(\begin{smallmatrix} 1 & 0 \\ 0 & -1 \end{smallmatrix})|(\frac{1}{2}, \frac{1}{6})\}$ and $\sigma = \{(\begin{smallmatrix} 1 & 0 \\ 0 & -1 \end{smallmatrix})|(0, \frac{2}{3})\}$ and we have $T_y(a\mathcal{T})(\sigma) = (\sigma)(a\mathcal{T})$, where T_y denotes a translation along the y-axis by one unit vector. The anticommutation of the symmetries $a\mathcal{T}$ and σ along the Brillouin zone boundary in the y-direction gives the two-fold degeneracy along C-Y.

Note that a previous study on $NiPS_3$ has also observed the magnetization axis slightly tilted away from the honeycomb plane[58]. This will not change the symmetry properties and our arguments about the extra degeneracies still hold.

E. Effect of photoexcitation on the magnon energy

In the main text, we discuss the dynamics of the photoexcitation mechanism in which the pump photon energy is in the vicinity of the spin–orbit-entangled excitons. Here, we provide a rough estimate of the scale of the magnon energy redshift. The magnon gap is given by $\epsilon_0 = \sqrt{-4D_x(D_z + 6J_3) + 4D_x^2}S$ in the simplified model of the previous section. The reduction in the magnetization is $\Delta S = \Delta n/N$, where Δn is the photo-generated exciton density, which is proportional to the absorbed pump laser fluence, and N is the total number of Ni sites. Therefore, qualitatively the redshift of the magnon energy is given by $\Delta\epsilon_0/\epsilon_0 = \Delta n/N$. Note that we have neglected the potential change in the spin-spin exchange coupling and the quantum fluctuation of spin-1/2, so the result we obtain above only provides a qualitative understanding of the linear dependence of the magnon energy on the absorbed fluence.

References

[1] Furman, S. A. & Tikhonravov, A. V. *Basics of Optics of Multilayer Systems* (Editions Frontières, Gif-sur-Yvette, 1992).

[2] Karlsen, P. & Hendry, E. Approximations used in the analysis of signals in pump-probe spectroscopy. Preprint at https://arXiv.org/abs/1902.08619 (2019).

[3] Beard, M. C., Turner, G. M. & Schmuttenmaer, C. A. Subpicosecond carrier dynamics in low-temperature grown GaAs as measured by time-resolved terahertz spectroscopy. *J. Appl. Phys.* **90**, 5915–5923 (2001).

[4] Ulbricht, R., Hendry, E., Shan, J., Heinz, T. F. & Bonn, M. Carrier dynamics in semiconductors studied with time-resolved terahertz spectroscopy. *Rev. Mod. Phys.* **83**, 543 (2011).

[5] Larsen, C., Cooke, D. G. & Jepsen, P. U. Finite-difference time-domain analysis of time-resolved terahertz spectroscopy experiments. *J. Opt. Soc. Am. B* **28**, 1308–1316 (2011).

[6] Kim, S. Y. *et al.* Charge-spin correlation in van der Waals antiferromagnet $NiPS_3$. *Phys. Rev. Lett.* **120**, 136402 (2018).

[7] Kang, S. *et al.* Coherent many-body exciton in van der Waals antiferromagnet $NiPS_3$. *Nature* **583**, 785–789 (2020).

[8] Kaindl, R. A., Carnahan, M. A., Hägele, D., Lövenich, R. & Chemla, D. S. Ultrafast terahertz probes of transient conducting and insulating phases in an electron–hole gas. *Nature* **423**, 734–738 (2003).

[9] Huber, R., Schmid, B. A., Shen, Y. R., Chemla, D. S. & Kaindl, R. A. Stimulated terahertz emission from intraexcitonic transitions in Cu_2O. *Phys. Rev. Lett.* **96**, 017402 (2006).

[10] Kuzmenko, A. B. Kramers–Kronig constrained variational analysis of optical spectra. *Rev. Sci. Instrum.* **76**, 083108 (2005).

[11] Lane, C. & Zhu, J.-X. Thickness dependence of electronic structure and optical properties of a correlated van der Waals antiferromagnet $NiPS_3$ thin film. *Phys. Rev. B* **102**, 075124 (2020).

[12] Beard, M. C., Turner, G. M. & Schmuttenmaer, C. A. Transient photoconductivity in GaAs as measured by time-resolved terahertz spectroscopy. *Phys. Rev. B* **62**, 15764 (2000).

[13] Zielbauer, J. & Wegener, M. Ultrafast optical pump THz-probe spectroscopy on silicon. *Appl. Phys. Lett.* **68**, 1223–1225 (1996).

[14] Kampfrath, T., Perfetti, L., Schapper, F., Frischkorn, C. & Wolf, M. Strongly coupled optical phonons in the ultrafast dynamics of the electronic energy and current relaxation in graphite. *Phys. Rev. Lett.* **95**, 187403 (2005).

[15] Hall, R. N. Electron-hole recombination in germanium. *Phys. Rev.* **87**, 387 (1952).

[16] Shockley, W. & Read Jr, W. T. Statistics of the recombinations of holes and electrons. *Phys. Rev.* **87**, 835–842 (1952).

[17] Linnros, J. Carrier lifetime measurements using free carrier absorption transients. I. Principle and injection dependence. *J. Appl. Phys.* **84**, 275–283 (1998).

[18] Lui, K. P. H. & Hegmann, F. A. Fluence-and temperature-dependent studies of carrier dynamics in radiation-damaged silicon-on-sapphire and amorphous silicon. *J. Appl. Phys.* **93**, 9012–9018 (2003).

[19] Okamoto, H. *et al.* Photoinduced transition from Mott insulator to metal in the undoped cuprates Nd_2CuO_4 and La_2CuO_4. *Phys. Rev. B* **83**, 125102 (2011).

[20] Hendry, E., Wang, F., Shan, J., Heinz, T. F. & Bonn, M. Electron transport in TiO_2 probed by THz time-domain spectroscopy. *Phys. Rev. B* **69**, 081101(R) (2004).

[21] Dean, N. *et al.* Polaronic conductivity in the photoinduced phase of $1T$-TaS_2. *Phys. Rev. Lett.* **106**, 016401 (2011).

[22] Emin, D. *Polarons* (Cambridge Univ. Press, Cambridge, 2013).

[23] Piacentini, M., Khumalo, F. S., Olson, C. G., Anderegg, J. W. & Lynch, D. W. Optical transitions, XPS, electronic states in $NiPS_3$. *Chem. Phys.* **65**, 289–304 (1982).

[24] Braun, C. L. Singlet exciton-exciton interaction in anthracene. *Phys. Rev. Lett.* **21**, 215–219 (1968).

[25] Bergman, A. & Jortner, J. Photoconductivity of crystalline anthracene induced by tunable dye lasers. *Phys. Rev. B* **9**, 4560–4574 (1974).

[26] Catalano, I. M., Cingolani, A., Ferrara, M. & Minafra, A. Luminescence by exciton-exciton collision in GaSe. *Phys. Status Solidi B* **68**, 341–347 (1975).

[27] Lee, C. H. *et al.* Transient and steady-state photoconductivity of a solid C_{60} film. *Phys. Rev. B* **48**, 8506–8509(R) (1993).

[28] Sun, D. *et al.* Observation of rapid exciton–exciton annihilation in monolayer molybdenum disulfide. *Nano Lett.* **14**, 5625–5629 (2014).

[29] Kalashnikova, A. M. *et al.* Impulsive generation of coherent magnons by linearly polarized light in the easy-plane antiferromagnet $FeBO_3$. *Phys. Rev. Lett.* **99**, 167205 (2007).

[30] Kimel, A. V. *et al.* Ultrafast non-thermal control of magnetization by instantaneous photomagnetic pulses. *Nature* **435**, 655–657 (2005).

[31] Hase, M., Kitajima, M., Constantinescu, A. M. & Petek, H. The birth of a quasiparticle in silicon observed in time–frequency space. *Nature* **426**, 51–54 (2003).

[32] Almeida, V. R., Barrios, C. A., Panepucci, R. R. & Lipson, M. All-optical control of light on a silicon chip. *Nature* **431**, 1081–1084 (2004).

[33] Rivas, J. G., Sánchez-Gil, J. A., Kuttge, M., Bolivar, P. H. & Kurz, H. Optically switchable mirrors for surface plasmon polaritons propagating on semiconductor surfaces. *Phys. Rev. B* **74**, 245324 (2006).

[34] Haug, H. & Schmitt-Rink, S. Basic mechanisms of the optical nonlinearities of semiconductors near the band edge. *J. Opt. Soc. Am. B* **2**, 1135–1142 (1985).

[35] Ishioka, K. & Misochko, O. V. Coherent lattice oscillations in solids and their optical control. In *Progress in Ultrafast Intense Laser Science*, 23–46 (Springer, 2010).

[36] Garrett, G. A., Whitaker, J. F., Sood, A. K. & Merlin, R. Ultrafast optical excitation of a combined coherent-squeezed phonon field in $SrTiO_3$. *Opt. Express* **1**, 385–389 (1997).

[37] Liao, B., Maznev, A. A., Nelson, K. A. & Chen, G. Photo-excited charge carriers suppress sub-terahertz phonon mode in silicon at room temperature. *Nat. Commun.* **7**, 13174 (2016).

[38] Zeiger, H. J. *et al.* Theory for displacive excitation of coherent phonons. *Phys. Rev. B* **45**, 768–778 (1992).

[39] Stevens, T. E., Kuhl, J. & Merlin, R. Coherent phonon generation and the two stimulated Raman tensors. *Phys. Rev. B* **65**, 144304 (2002).

[40] Ruello, P. & Gusev, V. E. Physical mechanisms of coherent acoustic phonons generation by ultrafast laser action. *Ultrasonics* **56**, 21–35 (2015).

[41] Ishioka, K. *et al.* Ultrafast electron-phonon decoupling in graphite. *Phys. Rev. B* **77**, 121402(R) (2008).

[42] Khomskii, D. I. *Transition Metal Compounds* (Cambridge Univ. Press, Cambridge, 2014).

[43] Dean, M. P. M. *et al.* Ultrafast energy-and momentum-resolved dynamics of magnetic correlations in the photo-doped Mott insulator Sr_2IrO_4. *Nat. Mater.* **15**, 601–605 (2016).

[44] Afanasiev, D. *et al.* Ultrafast spin dynamics in photodoped spin-orbit Mott insulator Sr_2IrO_4. *Phys. Rev. X* **9**, 021020 (2019).

[45] Yang, J.-A., Pellatz, N., Wolf, T., Nandkishore, R. & Reznik, D. Ultrafast magnetic dynamics in insulating $YBa_2Cu_3O_{6.1}$ revealed by time resolved two-magnon Raman scattering. *Nat. Commun.* **11**, 2548 (2020).

[46] Balzer, K., Wolf, F. A., McCulloch, I. P., Werner, P. & Eckstein, M. Nonthermal melting of Néel order in the Hubbard model. *Phys. Rev. X* **5**, 031039 (2015).

[47] Gretarsson, H. *et al.* Raman scattering study of vibrational and magnetic excitations in $Sr_{2-x}La_xIrO_4$. *Phys. Rev. B* **96**, 115138 (2017).

[48] Battisti, I. *et al.* Universality of pseudogap and emergent order in lightly doped Mott insulators. *Nat. Phys.* **13**, 21–25 (2017).

[49] Chu, H. *et al.* Linear magnetoelectric phase in ultrathin $MnPS_3$ probed by optical second harmonic generation. *Phys. Rev. Lett.* **124**, 027601 (2020).

[50] Wildes, A. R., Roessli, B., Lebech, B. & Godfrey, K. W. Spin waves and the critical behaviour of the magnetization in $MnPS_3$. *J. Phys.: Condens. Matter* **10**, 6417 (1998).

[51] Lançon, D. *et al.* Magnetic structure and magnon dynamics of the quasi-two-dimensional antiferromagnet $FePS_3$. *Phys. Rev. B* **94**, 214407 (2016).

[52] Brec, R., Schleich, D. M., Ouvrard, G., Louisy, A. & Rouxel, J. Physical properties of lithium intercalation compounds of the layered transition-metal chalcogenophosphites. *Inorg. Chem.* **18**, 1814–1818 (1979).

[53] Grasso, V., Santangelo, S. & Piacentini, M. Optical absorption spectra of some transition metal thiophosphates. *Solid State Ion.* **20**, 9–15 (1986).

[54] Banda, E. J. K. B. Optical absorption of $FePSe_3$ in the near infrared, visible, and near ultraviolet regions. *Phys. Status Solidi B* **138**, K125–K129 (1986).

[55] Grasso, V., Neri, F., Perillo, P., Silipigni, L. & Piacentini, M. Optical-absorption spectra of crystal-field transitions in $MnPS_3$ at low temperatures. *Phys. Rev. B* **44**, 11060–11066 (1991).

[56] Joy, P. A. & Vasudevan, S. Optical-absorption spectra of the layered transition-metal thiophosphates MPS_3 (M=Mn, Fe, and Ni). *Phys. Rev. B* **46**, 5134–5141 (1992).

[57] Gnatchenko, S. L., Kachur, I. S., Piryatinskaya, V. G., Vysochanskii, Y. M. & Gurzan, M. I. Exciton-magnon structure of the optical absorption spectrum of antiferromagnetic $MnPS_3$. *Low Temp. Phys.* **37**, 144–148 (2011).

[58] Kim, K. *et al.* Suppression of magnetic ordering in XXZ-type antiferromagnetic monolayer $NiPS_3$. *Nat. Commun.* **10**, 345 (2019).

[59] Lançon, D., Ewings, R. A., Guidi, T., Formisano, F. & Wildes, A. R. Magnetic exchange parameters and anisotropy of the quasi-two-dimensional antiferromagnet $NiPS_3$. *Phys. Rev. B* **98**, 134414 (2018).
